# The Systemic Immune-Inflammation Index May Predict the Coronary Slow Flow Better Than High-Sensitivity C-Reactive Protein in Patients Undergoing Elective Coronary Angiography

**DOI:** 10.1155/2022/7344639

**Published:** 2022-11-09

**Authors:** Kurtulus Karauzum, Irem Karauzum, Kaan Hanci, Dogus Gokcek, Beyzanur Gunay, Hussain Bakhshian, Tayfun Sahin, Ertan Ural

**Affiliations:** Kocaeli University School of Medicine, Department of Cardiology, Izmit, Kocaeli, Turkey

## Abstract

**Methods:**

A total of 197 patients (102 patients with CSF; 95 patients with normal coronary flow) were included in this retrospective study. Clinical and angiographic characteristics of patients were obtained from hospital records.

**Results:**

Patients with CSF had higher SII, neutrophil-to-lymphocyte ratio (NLR), platelet-to-lymphocyte (PLR), and high-sensitivity C-reactive protein (hsCRP) levels compared with the control group. Body mass index (*p*=0.022, OR 1.151, 95% CI 1.121–1.299), low-density lipoprotein (*p*=0.018, OR 1.028, 95% CI 1.005–1.052), hsCRP (*p*=0.044, OR 1.161, 95% CI 1.004–1.343), and SII (*p* < 0.001, OR 1.015, 95% CI 1.003–1.026) were independent predictors of CSF in the multivariable analysis. The optimal cutoff value of SII in predicting CSF was >877 in ROC curve analysis (*p* < 0.001, AUC = 0.892, 95% CI 0.848–0.936). This cutoff value of SII predicted the CSF with a sensitivity of 71.5% and specificity of 92.4%. Spearman correlation analysis showed a positive correlation between the mean TFC value and PLR, NLR, hsCRP, and SII.

**Conclusions:**

SII may be used as a better indicator for the prediction of CSF than hsCRP.

## 1. Introduction

Coronary slow flow (CSF) is an angiographic finding characterized by delayed distal vessel opacification of nonobstructive epicardial coronary arteries [1]. CSF is not uncommon and its incidence is reported to range between 1% and 3% in different populations undergoing coronary angiography [2, 3]. CSF has long been known by interventional cardiologists and usually appears in young male smokers [4]. Patients with CSF can present with important clinical manifestations such as cardiac dysfunction, recurrent angina pectoris, acute coronary syndromes (ACS), arrhythmias, and even sudden death [5, 6].

The definite underlying mechanism of CSF is not yet fully understood. The authors have suggested that pathophysiology of CSF may be complex and multifactorial [7]. CSF had been linked to small vessel disease when it was first described [8]. This was supported by showing microvascular damage in a few histopathological studies of patients with CSF undergoing endomyocardial biopsy [9, 10]. Microvascular vasomotor impairment and endothelial dysfunction have been shown to be important contributing factors of CSF [11, 12]. Additionally, early diffuse atherosclerosis was emphasized in subsequent studies using coronary intravascular imaging [13, 14]. Besides, chronic inflammation has been considered to play a major role in the development of CSF [15, 16]. In this regard, several inflammation-based biomarkers including high-sensitivity C-reactive protein (hsCRP), albumin, uric acid, serum soluble adhesion molecules, and peripheral blood cells have been reported to be associated with CSF [17–21]. The systemic immune-inflammation index (SII) is a relatively novel inflammatory indicator derived from a combination of neutrophil, platelet, and lymphocyte counts [22]. SII was first developed to predict clinical outcomes and prognosis in gastrointestinal system malignancies [22, 23]. Subsequently, the clinical importance of SII was demonstrated in various cardiovascular diseases such as ACS, heart failure (HF), coronary artery disease (CAD), contrast-induced nephropathy (CIN), and aortic stenosis [24–28]. However, the relationship between the SII and CSF remains unclear. Therefore, our study aimed to investigate whether there is an association between the SII and CSF in patients undergoing diagnostic elective coronary angiography.

## 2. Patients and Methods

### 2.1. Study Population

**Figure 1:**
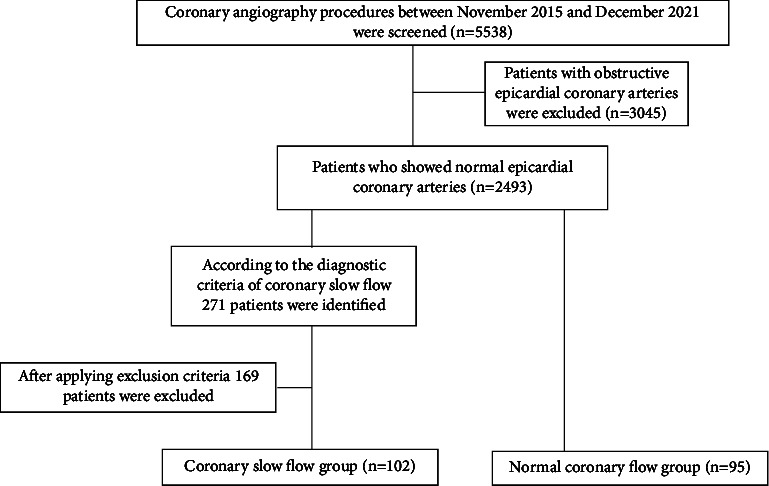
The flowchart diagram showing study patients enrollment.

A total of 5538 patients who underwent elective diagnostic coronary angiography between November 2015 and December 2021 at our catheterization laboratory were retrospectively evaluated. All screened patients had typical chest pain or angina-equivalent symptoms with a positive noninvasive test or a high-risk clinic without a positive noninvasive test. Patients with obstructive coronary artery disease were excluded after initial evaluation (*n* = 3045), and the remaining patients with nonobstructive epicardial coronary arteries were reassessed (*n* = 2493). A total of 271 patients were identified according to the diagnostic criteria of CSF. The exclusion criteria were the history of ACS, percutaneous coronary intervention (PCI) and/or surgical bypass (*n* = 41), hypotension during the procedure (*n* = 4), coronary artery spasm (*n* = 5), coronary ectasia or aneurysm (*n* = 8), chronic or acute HF (*n* = 21), significant valvular heart disease (*n* = 9), documented arrhythmias (*n* = 10), severe peripheral artery disease or cerebrovascular disease (*n* = 11), acute or chronic infection (*n* = 8), renal or hepatic impairment (*n* = 19), hematological diseases (*n* = 4), severe chronic obstructive pulmonary disease (*n* = 8), malignancies (*n* = 3), chronic inflammatory diseases (*n* = 8), chronic use of steroids or nonsteroidal anti-inflammatory drugs, and anticoagulant therapy (*n* = 10). After applying exclusion criteria, the remaining 102 patients with CSF and normal epicardial coronary arteries were enrolled in the study as the CSF group. Among the 2493 patients with nonobstructive coronary arteries, 95 patients with normal coronary flow who met the exclusion criteria were included as the control group. In total, 197 patients were included in the study, 102 of whom were patients with CSF without any stenosis and 95 of whom had normal coronary arteries and normal coronary flow (Figure 1).

All data were obtained from the hospital database records and patient files at the time of coronary angiography procedure. Demographic data and medical history such as age, sex, body mass index (BMI), diabetes, hypertension, hyperlipidemia, smoking status, family history of CAD, and medical therapy were collected. Vital signs including blood pressure and heart rate were recorded.

The study protocol was approved by the local ethical committees (GOKAEK-2022/04.21; Project No.: 2022-65). This study was conducted in accordance with the ethical principles of the Declaration of Helsinki.

### 2.2. Coronary Angiography

Coronary angiography was performed using the standard Judkins technique with a Siemens imaging system. Angiographic views were obtained in the left and right oblique planes with caudal and cranial angulations to show the coronary arteries. The views were recorded at a film rate of 30 frames per second. Nonionic low-osmolar contrast medium (Iohexol, Omnipol 300 mg I/ml; Polifarma, Istanbul, Turkey) was used in all procedures. CSF was determined using the thrombolysis in myocardial infarction (TIMI) frame count which was described by Gibson and colleagues [29]. This quantitative approach is the most used method in the literature and assesses the number of cine frames required for the opacification of the distal coronary vessel vasculature [29]. The TIMI frame count (TFC) of coronary arteries was evaluated by two experienced interventionalists who were blinded to the clinical features of the patients. Frames, where the contrast agent first entered the coronary artery and reached the distal landmark, were determined as the first and final frames, respectively. The first frame was noted when >70% of the coronary vessel lumen was opacified using antegrade filling. The distal landmarks of final frames were distal bifurcation (“whales tail”) for the left anterior descending (LAD) artery, the most distal bifurcation of the obtuse marginal branch for circumflex (Cx) artery, and the first branch of the posterolateral segment for the right coronary artery (RCA) [30, 31]. The TFC for the LAD and Cx arteries was evaluated in the right anterior oblique projection with caudal angulation and RCA in the left anterior oblique projection with cranial angulation [31]. The cutoff values of the TFC for the normal filling of epicardial coronary arteries were 22.1 ± 4.1 for the Cx artery and 20.4 ± 3.1 for the RCA [30, 31]. The TFC for the LAD artery was divided by 1.7 to obtain the corrected TFC (cTFC) value because the course of the LAD artery is usually longer than RCA and Cx artery and cTFC for the LAD artery was 21.1 ± 1.5 frames [30, 31]. The mean TFC value was calculated from the mean value of the frame counts of the RCA, LAD, and Cx arteries. Any TFC value greater than two standard deviations from these published thresholds was accepted as a diagnosis of “CSF” [30–32].

### 2.3. Blood Sampling

All venous blood samples for measurements were drawn after a 12 hours fasting period 24 hours before the procedure. These samples were taken into standard tubes containing EDTA for complete blood count and analyzed using an automated blood cell counter within 30 min (Symex K-1000; Kobe, Japan). Biochemical analyses, including serum creatinine, lipid profile, and hsCRP levels, were performed using standard laboratory techniques (Roche Diagnostic Modular Systems; Tokyo, Japan). The SII was calculated using the following formula: (neutrophil count × platelet count)/lymphocyte count. NLR and PLR were calculated from the neutrophil count/lymphocyte count and platelet count/lymphocyte count, respectively. The estimated glomerular filtration rate (eGFR) was calculated using the Modification of Diet in Renal Disease formula [33].

### 2.4. Statistical Analysis

SPSS, version 20.0 software (IBM Inc., Chicago, Illinois, USA), was used for all analyses. The Kolmogorov−Smirnov test was used to assess the normality of the distribution of continuous variables. Continuous variables are presented as mean ± standard deviation or median and interquartile range according to the distribution pattern. Categorical variables are presented as numbers, percentages, or proportions. The chi-squared test was used to compare the proportions of the groups and categorical variables. Continuous variables with normal distributions were compared using the independent sample *t*-test, and continuous variables with non-normal distributions were compared using the Mann−Whitney *U* test. All analyses were two-sided and accepted significant at a value of *p* < 0.05. Demographic, clinical, laboratory, and angiographic characteristics were compared between the patients with CSF and normal coronary flow. Variables that demonstrated significant differences (*p* < 0.05) were evaluated as potential predictors in the univariate analysis. To avoid a multicollinearity problem, NLR, PLR, and other indices including neutrophils, lymphocytes, and platelets were not added to the logistic regression analysis. Variables associated with CSF on univariate analysis (*p* < 0.05) were included in the multivariable analysis and the results were shown as odds ratios (OR) with 95% confidence intervals (CI). Receiver operating characteristic (ROC) curve analysis was performed to identify the optimal cutoff values of the SII and hsCRP for the predicting CSF. The Spearman test was used for the correlation analysis.

## 3. Results

A total of 197 patients (mean age 56.8 ± 10.3 years, 64.5% male) with CSF and normal coronary flow were included in the study. Baseline demographic and clinical characteristics of the study population are presented in Table 1. The mean age of patients with CSF was lower than patients with normal coronary flow, but it was not statistically significant (55.7 ± 9.9 vs. 58.1 ± 10.6, *p*=0.058). The ratio of male gender was significantly higher in patients with CSF than those with normal coronary flow (74.3% vs. 53.3%, *p*=0.002). The patients with CSF were more overweight compared with controls. There was no difference between the two groups in terms of heart rate and systolic and diastolic blood pressures. The frequencies of hypertension, hyperlipidemia, and family history of CAD were similar in both groups. The patients with CSF were more diabetic and current smoker compared with normal coronary flow patients (*p*=0.026 and *p*=0.009, respectively).

Angiographic characteristics and laboratory results of the study patients are shown in Table 2. Patients with CSF had significantly higher TFC values for Cx artery (36.2 ± 4.7 vs. 20.1 ± 3.1, *p* < 0.001) and RCA (36.6 ± 5.5 vs. 18.9 ± 2.2, *p* < 0.001) and cTFC values for LAD artery (39.3 ± 4.9 vs. 19.9 ± 2.9, *p* < 0.001) than control subjects. Baseline eGFR and hemoglobin levels were similar between the two groups. The patients with CSF had markedly elevated LDL cholesterol levels compared with normal coronary flow patients (149.6 ± 34.9 vs. 134.7 ± 21.6, *p*=0.001). While lymphocyte count was lower (1.75 ± 0.71 vs. 2.47 ± 0.77, *p* < 0.001), neutrophil count (9.01 ± 4.17 vs. 4.69 ± 1.72, *p* < 0.001), platelet count (267 ± 72 vs. 184 ± 44, *p* < 0.001), PLR (150 (112–232) vs. 75 (59–101), *p* < 0.001), NLR (4.69 (3.34–7.89) vs. 1.86 (1.45–2.23), *p* < 0.001), hsCRP (4.4 (1.5–11.0) vs. 1.1 (0.8–2.2), *p* < 0.001), and SII (1313 (773–2066) vs. 539 (254–914), *p* < 0.001) were higher in patients with CSF.

The univariate and multivariable logistic regression analyses are presented in Table 3. Variables including male gender (*p*=0.002, OR 2.535, 95% CI 1.392–4.616), diabetes (*p*=0.028, OR 2.042, 95% CI 1.081–3.855), BMI (*p*=0.048, OR 1.070, 95% CI 1.001–1.145), smoking (*p*=0.010, OR 2.121, 95% CI 1.200–3.750), LDL cholesterol (*p*=0.001, OR 1.019, 95% CI 1.007–1.031), hsCRP (*p* < 0.001, OR 1.361, 95% CI 1.211–1.531), and SII (*p* < 0.001, OR 1.007, 95% CI 1.005–1.010) showed significant differences and were associated with CSF in the univariate analysis. In the multivariable analysis, BMI (*p*=0.022, OR 1.151, 95% CI 1.021–1.299), LDL cholesterol (*p*=0.018, OR 1.028, 95% CI 1.005–1.052), hsCRP (*p*=0.044, OR 1.161, 95% CI 1.004–1.343), and SII (*p* < 0.001, OR 1.015, 95% CI 1.003–1.026) were found to be independent predictors of CSF. Spearman correlation analysis showed a positive correlation between the mean TFC value and the PLR, NLR, hsCRP, and SII (Table 4).

**Figure 2:**
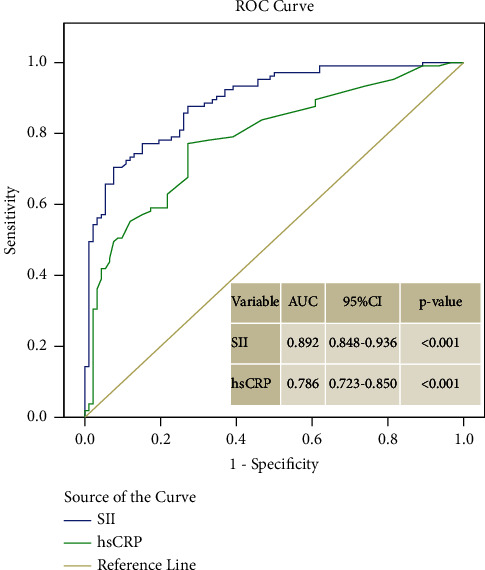
Receiver operating characteristic (ROC) curve analysis of systemic immune-inflammation index (SII) and high-sensitivity C-reactive protein for the prediction of coronary slow flow.

The optimal cutoff value of SII in predicting CSF was >877 with an area under the curve (AUC) in the ROC curve analysis (*p* < 0.001, AUC = 0.892, 95% CI 0.848–0.936). This cutoff value of SII predicted the development of CIN with a sensitivity of 71.5% and specificity of 92.4%. In addition, the optimal cutoff value of hsCRP was >1.4 with a sensitivity of 77.1% and specificity of 72.8% (*p* < 0.001, AUC = 0.786, 95% CI 0.723–0.850) (Figure 2).

## 4. Discussion

To the best of our knowledge, this is the first study to report a relationship between the SII and CSF. In the present study, BMI, LDL cholesterol, hsCRP, and SII were found to be independent predictors of CSF. Also, PLR, NLR, hsCRP, and SII showed a positive correlation with mean TFC values.

Near five decades ago, Tambe et al. first described the hemodynamic and clinical characteristics of a small patient group with chest pain and delayed distal vasculature opacification of nonobstructive epicardial coronary arteries on selective coronary angiography [8]. These authors suggested that small vessel disease could be the main pathophysiology of this important angiographic entity which was called CSF [8]. In addition to small-vessel disease, several possible hypotheses related to CSF have been proposed to date. Beltrame et al. revealed that the coronary flow reserve, which is an important indicator of coronary microvasculature function, is impaired in patients with SCF, and TFC values have shown a positive correlation with coronary flow reserve [33]. Also, it has been shown that coronary blood flow increases with calcium *T*-channel blocker treatment, which may reflect the presence of a component of microvascular spasms in the pathogenesis of CSF [34]. Sezgin et al. showed impaired endothelium-dependent vasodilatation in patients with CSF, although traditional major cardiovascular risk factors were absent [35]. The anatomical characteristic of the coronary arteries is a notable observation in the CSF which is more prevalent in patients with higher tortuosity and more distal branches of their coronary arteries [36].

Chronic inflammation has a well-known pivotal role in the development of cardiovascular diseases and it is associated with all stages of atherosclerosis, from initiation through progression [37]. In this regard, Turhan et al. showed that serum plasma soluble adhesion molecules such as intercellular adhesion molecule-1, vascular cell adhesion molecule-1, and *E*-selectin were markedly higher in patients with CSF than in controls [20]. Li et al. revealed that serum plasma concentrations of interleukin-6 and CRP levels were increased in patients with CSF [38]. Similarly, Barutcu et al. reported that patients with CSF had significantly elevated hsCRP levels compared with the control group [17]. In recent years, various indices derived from neutrophil, lymphocyte, and platelet counts have been increasingly developed to determine the relationship between inflammatory status and cardiovascular conditions. Among them, NLR and PLR, which are two of the most widely used indices, have been demonstrated to be associated with ACS, chronic or acute HF, CAD, valvular heart disease, and hypertension, as well as CSF [39–41]. The SII is defined as a relatively novel index based on the counts of neutrophil, platelet, and lymphocyte [22]. It has been also suggested that as the SII includes three different cells, it may better reflect the inflammatory status than other indices, which are typically based on two cells such as NLR and PLR or the lymphocyte-to-monocyte ratio. The first study on SII reported that it strongly predicted poor prognosis in several types of gastrointestinal and other systems malignancies [42]. Following these studies, the association between the SII and cardiovascular diseases has been increasingly investigated. Candemir et al. reported that SII may be used as a risk factor for CAD and a more accurate predictor of CAD severity than NLR and PLR [43]. In a study by Yang et al., higher SII levels were associated with an increased risk of clinical outcomes including nonfatal acute myocardial infarction, nonfatal stroke, HF-related hospitalization, and cardiac death [44]. A fractional flow reserve (FFR) study concluded that the SII was higher in patients with established functionally significant coronary artery stenosis [26]. This study also suggested that SII was superior to NLR and PLR in predicting the functional significance of moderate coronary lesions in the FFR examination [26]. Another study reported that a higher SII value may be associated with a greater risk of more severe pulmonary embolism [45]. Esenboga et al. demonstrated that SII was significantly higher in patients who developed a no-reflow phenomenon during primary PCI and that SII was an independent predictor of the no-reflow phenomenon in these patients [46]. A study by Erdogan et al. reported that SII is a useful indicator for predicting severe aortic stenosis [28]. They also showed that the SII was correlated with the aortic valve area and the mean aortic transvalvular gradient [28]. Our previous study demonstrated a relationship between the SII and CIN development in patients with ST-segment elevation myocardial infarction who underwent primary PCI [27]. We found that a higher SII was a predictive indicator of CIN, and the SII might be a better marker than NLR and PLR in predicting CIN [27]. Similar to all these studies, we recently found that patients with CSF had higher SII levels compared with coronary normal flow patients, as well as higher BMI, LDL cholesterol, and hsCRP. These parameters were independent predictors of CSF in our study population. Previous studies have shown that BMI, LDL cholesterol, and hsCRP are associated with CSF similar to our study [4, 7, 17, 30, 47]. ROC curve analysis reveals that SII might be a better indicator than hsCRP in patients with CSF. There was a positive correlation between the mean TFC and PLR, NLR, hsCRP, and SII.

### 4.1. Study Limitations

There are some limitations of the present study. This was a single-center, retrospective, and observational study with a relatively small number of patients. As we do not routinely exam the albumin, uric acid levels in patients undergoing elective coronary angiography could not be investigated in our study. Although it was statistically nearly significant, previous well-known indicators of CSF such as smoking, male gender, and younger age could not be shown to be independent predictors of CSF in the multivariable analysis due to the possible relatively small number of patients in the study. Additionally, clinical outcomes of the study population could not be evaluated in the follow-up. Therefore, large scale prospective randomised-controlled studies are needed to support the relationship between SII and CSF.

## 5. Conclusions

The SII is a relatively novel inflammation-based index derived from neutrophil, platelet, and lymphocyte counts, which has been shown to predict clinical outcomes in many malignancies and cardiovascular diseases [22–28, 48]. Our study demonstrated that patients with CSF had higher SII, BMI, LDL cholesterol, and hsCRP levels than patients with normal coronary flow. SII, hsCRP, BMI, and LDL were also found to be associated with CSF. We consider that SII is one of the parameters of routine CBC, which is reliable, inexpensive, and easily calculated; it can be suggested that the SII may be used in the prediction of CSF. However, future studies are needed to confirm these results and clearly disclose the pathophysiologic role of SII in CSF.

## Figures and Tables

**Table 1:** Baseline clinical characteristics of the patients with coronary slow flow and normal coronary flow.

Characteristics	All patients	CSF	Normal coronary flow	*P* value
	*n* = 197	*n* = 105	*n* = 92	
Age (years)	56.8 ± 10.3	55.7 ± 9.9	58.1 ± 10.6	0.058
Male, *n* (%)	127 (64.5%)	78 (74.3%)	49 (53.3%)	0.002
BMI, kg/m^2^	27.4 ± 4.3	28.1 ± 3.9	26.8 ± 4.7	0.019
SBP, mmHg	124.3 ± 20.5	126.7 ± 21.9	121.5 ± 18.4	0.116
DBP, mmHg	75.9 ± 11.8	76.8 ± 12.1	74.9 ± 11.5	0.407
Heart rate, bpm	76 ± 15	77 ± 13	75 ± 16	0.121

Comorbidities, *n* (%)				
Hypertension	84 (42.6%)	46 (43.8%)	38 (41.3%)	0.723
Diabetes mellitus	58 (29.4%)	38 (36.2%)	20 (21.7%)	0.026
Hyperlipidemia	60 (30.5%)	37(35.2%)	23 (25.0%)	0.119
Current smoker	103 (52.3%)	64 (61.0%)	39 (42.3%)	0.009
Family history of CAD	46 (23.4%)	24 (22.9%)	22 (23.9%)	0.861

Medical therapy on admission, *n* (%)				
ACE-*i* or ARB	73 (37.1%)	38 (36.2%)	35 (38.0%)	0.788
CCB	16 (8.1%)	9 (8.6%)	7 (7.6%)	0.805
Beta-blocker	16 (8.1%)	8 (7.6%)	8 (8.7%)	0.783
Statin	36 (18.3%)	16 (15.2%)	20 (21.7%)	0.239
Aspirin	44 (22.3%)	23 (21.9%)	21 (22.8)	0.877

ACE-*i*, angiotensin converting enzyme inhibitor; ARB, angiotensin receptor blocker; BMI, body mass index; BPM = Beat per minute; CAD = Coronary artery disease; CCB = Calcium channel blocker; CSF = Coronary slow flow; DBP = Diastolic blood pressure; SBP = Systolic blood pressure.

**Table 2:** Angiographic characteristics and laboratory results of the patients with coronary slow and normal coronary flow.

Variable	All patients (*n* = 197)	CSF (*n* = 105)	Normal coronary flow (*n* = 92)	*P* value
Coronary flow rate, frame counts				
Corrected TFC of LAD artery	30.2 ± 10.6	39.3 ± 4.9	19.9 ± 2.9	<0.001
TFC of Cx artery	28.6 ± 9.1	36.2 ± 4.7	20.1 ± 3.1	<0.001
TFC of RCA	28.3 ± 9.9	36.6 ± 5.5	18.9 ± 2.2	<0.001
Mean TFC	29.1 ± 9.3	37.4 ± 3.2	19.6 ± 1.6	<0.001

Laboratory findings				
eGFR, mL/min/1.73 m^2^	94.7 ± 24.8	93.6 ± 15.7	95.9 ± 36.2	0.514
Hemoglobin, g/dl	13.9 ± 1.7	13.9 ± 1.8	13.8 ± 1.6	0.270
LDL cholesterol, mg/dl	142.7 ± 30.4	149.6 ± 34.9	134.7 ± 21.6	0.001
Neutrophil count, (×10^3^/ml)	6.99 ± 3.90	9.01 ± 4.17	4.69 ± 1.72	<0.001
Lymphocyte count, (×10^3^/ml)	2.08 ± 0.82	1.75 ± 0.71	2.47 ± 0.77	<0.001
Platelet count, (×10^3^/ml)	228 ± 73	267 ± 72	184 ± 44	<0.001
PLR, median (IQR)	106 (74–154)	150 (112–232)	75 (59–101)	<0.001
NLR, median (IQR)	2.78 (1.86–5.06)	4.69 (3.34–7.89)	1.86 (1.45–2.23)	<0.001
hsCRP, mg/dl, median (IQR)	1.9 (1.1–5.7)	4.4 (1.5–11.0)	1.1 (0.8–2.2)	<0.001
SII, (×10^3^/ml), median (IQR)	748 (443–1388)	1313 (773–2066)	539 (254–914)	<0.001

CSF, coronary slow flow; eGFR, estimated glomerular filtration rate; hsCRP, high-sensitivity C-reactive protein; IQR, interquartile range; LAD, left anterior descending; Cx, circumflex; LDL, low-density lipoprotein; NLR, neutrophil-to-lymphocyte ratio; PLR, platelet-to-lymphocyte ratio; RCA, right coronary artery; SII, systemic immune-inflammation index; TFC, thrombolysis in myocardial infarction frame count.

**Table 3:** Univariate and multivariable logistic regression analyses for predicting coronary slow flow.

Variable	Univariate analysis		Multivariable analysis	
	OR (95% CI)	*P* value	OR (95% CI)	*P* value
Male gender	2.535 (1.392–4.616)	0.002	2.746 (0.864–8.730)	0.087
Diabetes	2.042 (1.081–3.855)	0.028	2.738 (0.816–9.192)	0.103
BMI	1.070 (1.001–1.145)	0.048	1.151 (1.021–1.299)	**0.022**
Smoking	2.121 (1.200–3.750)	0.010	2.837 (0.894–9.001)	0.077
LDL	1.019 (1.007–1.031)	0.001	1.028 (1.005–1.052)	**0.018**
hsCRP	1.361 (1.211–1.531)	<0.001	1.161 (1.004–1.343)	**0.044**
SII	1.007 (1.005–1.010)	<0.001	1.015 (1.003–1.026)	**<0.001**

BMI, body mass index; hsCRP, high-sensitivity C-reactive protein; LDL, low-density lipoprotein; OR, odds ratio; SII, systemic immune-inflammatory index.

**Table 4:** Spearman's rho correlation analysis between the mean TFC and inflammation-based indicators.

Indicator	Mean TFC	
	*r* value	*P* value
PLR	0.484	<0.001
NLR	0.600	<0.001
hsCRP	0.671	<0.001
SII	0.725	<0.001

hsCRP, high sensitivity C-reactive protein; NLR, neutrophil-to-lymphocyte ratio; PLR, platelet-to-lymphocyte ratio; SII, systemic immune-inflammation index; TFC, TIMI frame count.

## Data Availability

The datasets generated during and/or analyzed during the current study are not publicly available, but are available from the corresponding author upon request.

## Abbreviations

ACS: Acute coronary syndrome
AUC: Area under the curve
BMI: Body mass index
CAD: Coronary artery disease
CI: Confidence interval
CIN: Contrast-induced nephropathy
CSF: Coronary slow flow
Cx: Circumflex
FFR: Fractional flow reserve
HF: Heart failure
hsCRP: High-sensitivity C-reactive protein
LAD: Left anterior descending
LDL: Low-density lipoprotein
NLR: Neutrophil-to-lymphocyte ratio
OR: Odds ratio
PCI: Percutaneous coronary intervention
PLR: Platelet-to-lymphocyte ratio
RCA: Right coronary artery
ROC: Receiver operating characteristic
SII: Systemic immune-inflammation index
TFC: Thrombolysis in myocardial infarction frame count
TIMI: Thrombolysis in myocardial infarction.
